# The Out-Of-Plane Compression Behavior of In Situ Ethylene Vinyl Acetate (EVA)-Foam-Filled Aluminum Honeycomb Sandwich Structures

**DOI:** 10.3390/ma16155350

**Published:** 2023-07-30

**Authors:** Tuğba Selcen Atalay Kalsen, Hakan Burak Karadağ, Yasin Ramazan Eker

**Affiliations:** 1 Department of Metallurgical and Materials Engineering, Köyceğiz Campus, Necmettin Erbakan University, Meram, Konya 42090, Turkey; tsatalay@erbakan.edu.tr; 2 Research and Application Center of Science and Technology (BİTAM), Köyceğiz Campus, Necmettin Erbakan University, Meram, Konya 42090, Turkey; yeker@erbakan.edu.tr

**Keywords:** aluminum honeycomb, out-of-plane compression, ethylene vinyl acetate foam, in situ foaming, springback

## Abstract

In this study, the mechanical behavior of aluminum honeycomb (AHC) sandwich structures filled with ethylene vinyl acetate copolymer (EVA) foam in situ under out-of-plane compression loading was investigated experimentally. Both non-filled and EVA-foam-filled sandwich specimens with three different AHC core cell sizes (5.20, 6.78, and 8.66 mm) were studied to correlate the foam-filling effect with a key structural parameter. The results showed that compression characteristic properties such as peak stress, plateau stress, and absorbed energy per unit volume of the sandwich structure increased with EVA foam filling. The structure showed high recoverability when the compression loading was removed due to the viscoelastic nature of EVA foam. Cored EVA sandwich with 8.66 mm AHC cell size was recovered at 44% of the original thickness. This result promises groundbreaking applications such as impact-resistant and self-healing structures. The microstructures were also observed using scanning electron microscopy (SEM) to investigate the failure and the recoverability mechanisms.

## 1. Introduction

Recently, the search for lightweight structural materials, which are substantial for critical application areas such as aviation, space, automotive, and railways, has been the driving force in the development of lightweight and stiff structures. The use of cellular solids such as honeycomb, metal foam, polymer foam, and their combinations, which can be produced in different configurations and have specific mechanical properties, especially in thin-walled structures, has become increasingly common [1,2,3,4,5]. The AHC, which has been used as a core material in sandwich structures, has undoubtedly been one of the most important members of this group. Its specific hexagonal shape ensures that the cell walls are arranged symmetrically and periodically in the maximum volume using the minimum amount of material, as seen in the honeycomb of bees in nature. Many theoretical and experimental studies have been conducted on the mechanical properties and damage mechanism of AHCs with characteristic geometric parameters such as cell size, wall thickness, and core thickness [6,7,8,9,10,11,12]. According to these studies, the mechanical property of high strength in the out-of-plane direction compared to the other two in-plane directions (longitudinal/ribbon and width/transverse to ribbon) due to their anisotropic nature has been demonstrated [8,13]. Moreover, certain characteristic compression properties, such as yield strength, load-bearing capacity, plateau stress, and densification point, that affect their usability in critical applications could be obtained from the mechanical behavior of an AHC under quasistatic out-of-plane loading.

Recently, reinforcement of cellular materials used as cores made of different base materials (aluminum [14,15,16], Nomex [17,18], polycarbonate [19], and paper [20]) with filling foams, as in thin-walled structures, has shown an increasing trend in the literature due to its improved specific mechanical properties. Nia and Sadeghi [14] stated that the mean crushing strength and energy-absorbing capacity of the AHC structures filled with polyurethane foam at different densities (16, 29, and 52 kg/m^3^) increased. Their experimental study also showed that the number of folds increased with the decrease in the half-wavelength of folds, with a more regular trend in PU-foam-filled honeycomb after out-of-plane compression [14]. Similarly, Mahmoudabadi and Sadighi [21] investigated the crushing properties of the PU-foam-filled AHC under quasistatic conditions via an out-of-plane compression test and investigated the dynamic conditions via a low-velocity impact test. They reported that the ratio of the peak load to the mean crushing load decreased in foam-filled samples and that the mean crushing stress increased with foam-filling [21]. Mozafari et al. [22] studied the in-plane compression behavior of a honeycomb structure filled with PU foam of different densities (65, 90, and 145 kg/m^3^), and they stated that the in-plane crushing strength and specific absorbed energy were significantly increased in the foam-filled AHC. Moreover, Liu et al. [23] investigated the mechanical properties of an AHC filled with expanded polypropylene (EPP) foam instead of polyurethane foam under quasistatic compression loadings in both in-plane and out-of-plane directions. Similarly, Zhang et al. [16] also used EPP-foam-filled honeycomb under dynamic conditions. Their results indicated that filling with the EPP foam increased the load-bearing capacity of the honeycomb, especially under lateral compression, and provided an improvement in the mean strength and initial peak strength under dynamic conditions due to the interaction between the aluminum cell wall and the foam [16,23]. Additionally, Mohamadi et al. [24] revealed that the energy absorption capacity of AHC structure increased when filling it with elastomeric polyurethane foam and emphasized that elastomeric foams could play an important role in energy absorption applications due to their damping properties and reversibility versus deformation.

Studies including foam-filled aluminum hexagonal structures have shown that filling provides reinforcement for the structure concerning its physical and mechanical aspects. However, the foaming process and polymer foam properties have become significant factors in the manufacturing of these structures and resultant mechanical properties, considering the interaction between the aluminum cell wall and the polymer foam. Polymer foams, except polyurethane, are not recommended for in situ foaming techniques used for filling hexagonal honeycomb structures. In one study, EPP foam pieces cut in hexagonal cell sizes were transferred to honeycomb cells after applying adhesive inside; thus, filled sandwich structures were obtained [16,23]. Such insertion techniques do not seem suitable for the mass production of large parts, even when assuming that geometrically perfect aluminum hexagonal structures are produced, and involve economic concerns as they involve extra processing steps. Consequently, the use of novel polymer foams for filling structures with critical physical and mechanical properties is becoming increasingly important, especially considering the in situ foaming process.

Ethylene vinyl acetate (EVA) copolymers, containing thermoplastic ethylene and elastomeric vinyl acetate blocks, are used in wide application areas, such as footwear, sports equipment, adhesive films, and cable coverings, owing to their variable properties mainly depending on their vinyl acetate content [25,26]. EVA is also easily processable due to its low softening temperature, and adding additives such as crosslinking and foaming agents can modify its physical and mechanical properties [27]. Moreover, EVA has a relatively low cost when evaluated for a similar polymer group such as thermoplastic polyurethane [27]. Foams made from EVA exhibit high impact resistance, vibration absorption capacity, and great recoverability characteristics after compression due to their viscoelastic nature [28,29,30]. Chang et al. [31] demonstrated that the impact absorption is better by EVA foam when compared with the same density EPP foam when used as cushioning materials for bulletproof plates. Moreover, Zhang et al. [32] stated that the compression set, which is inversely proportional to the elastic recovery characteristic after releasing the compression loadings of EVA foam, was significantly lower than that of elastomeric polyurethane. Therefore, EVA foam has great potential as a filler for reinforcing thin-walled structures due to its high energy-absorbing capability and providing a characteristic mechanical response under compressive loading.

The first purpose of this study was to fill ethylene vinyl acetate (EVA) foam into an AHC via an in situ thermochemical foaming process avoiding thermal and mechanical damage to aluminum cell walls. The second purpose of this study was to investigate the out-of-plane compression behavior of novel in situ EVA-foam-filled aluminum hexagonal sandwich structures considering the interaction between aluminum cell walls and polymer foam. The novelty of the study is the introduction of EVA copolymer within aluminum honeycomb via both in situ polycondensation and foaming. Moreover, the compression behavior of the EVA foam and the AHC was investigated as a sandwich structure with face sheets actually usable in application areas. The study also investigated the effect of cell size of both empty and foam-filled AHC and its failure mechanism after compression using detailed SEM analysis.

## 2. Materials and Methods

The AHC cores (Al 3005 H19) with different cell sizes, facing sheets (Al 5754), and polyurethane adhesives were supplied from 6Gen Panel Aerospace Shipbuilding Panel Industry Inc., Konya, Turkey. The properties of the core material such as cell size, thickness, and density (calculated according to Bitzer [33]) are given in Table 1.

Greenflex^®^ ML 50 Ethylene vinyl acetate copolymer (EVA) containing 19% vinyl acetate (0.941 g/cm^3^ density at RT, 83 °C melting point) was used as the raw material. Commercially available and widely used additives—azodicarbonamide (AZD) as a chemical blowing agent (97%, Kimteks), zinc oxide (ZnO) as an activator (99%, Melos A.Ş.), and dicumyl peroxide (DCP) as a chemical crosslinking agent (99%, Promagnus)—were used for the EVA foaming process. Toluene (Emsure^®^ Grade) used for homogeneously mixing EVA and additives via solution mixing method was purchased from Merck, Germany.

EVA granules were first dissolved in toluene, which was added at a 1:4 (EVA/Toluene) ratio calculated as g/mL at approximately its melting temperature (80 °C). The process was carried out until the dissolution of the whole EVA granule. AZD, ZnO, and DCP were added to the EVA solution as 3 wt%, 3 wt%, and 1 wt%, respectively. After evaporating the toluene, the mixture was introduced into the honeycomb by hand. To realize foaming, EVA mixture-filled HC in a square mold was heated at 160 °C for 1 h with PID-controlled heat plates under 20 kg·f.

Aluminum face sheets were bonded to the empty or EVA-filled honeycombs by polyurethane adhesive. During the process, PU adhesive mix was applied on each 5754 aluminum sheets at a constant thickness of 400 µm via a cylindrical baker film applicator. Afterwards, the sandwich panels were cured at 80 °C for 15 min.

The quasistatic compression tests were performed using the SHIMADZU AGS-X universal (SHIMADZU CORPORATION Analytical & Measuring Instruments Division Address: 1,NISHINOKYO-KUWABARACHO, NAKAGYO-KU,KYOTO,604-8511, JAPAN) testing machine at ambient temperature. The foam-filled HC and the unfilled HC sandwich panels were placed on a support platen and loaded axially with a movable crosshead at a rate of 1 mm/min. Three samples were tested for each parameter.

The energy efficiency parameter ($E_{f}$) was calculated according to [23]:

(1) $$E_{f}=\frac{\int_{0}^{\varepsilon a}\sigma \left(\varepsilon \right)d\varepsilon }{\sigma_{a}}$$

(2) $$\frac{dE_{f}\left(\varepsilon_{a}\right)}{d\varepsilon_{a}}=0\;0\leq \varepsilon_{a}\leq 1,$$

where $\varepsilon_{a}$ denotes the particular strain and $\sigma_{a}$ is the stress at the corresponding strain values. The strain at the maximum point of $E_{f}$ is the densification initiation strain (*ε_d_*) based on the energy efficiency method described by Avalle et al. [34], Tan et al. [35], and Li et al. [36].

The plateau stress ($\sigma_{pl}$) between the obtained collapse initiation strain ($\varepsilon_{c0}$) and the densification initiation strain (*ε_d_*) was calculated as described by Sun and Li [37]:

(3) $$\sigma_{pl}=\frac{\int_{\varepsilon_{c0}}^{\varepsilon_{d}}\sigma \left(\varepsilon \right)d\varepsilon }{\varepsilon_{d}-\varepsilon_{c0}}$$

Moreover, the plastic energy, which is absorbed (per unit volume) by the material, obtained from the area under the stress–strain curve between the collapse initiation strain ($\varepsilon_{c0}$) and the densification initiation strain (*ε_d_*), was calculated according to Equation (4) by Sun and Li [37]:

(4) $$U_{pl}=\int_{\varepsilon_{c0}}^{\varepsilon_{d}}\sigma \left(\varepsilon \right)d\varepsilon$$

where $U_{pl}$ denotes the plastic energy per unit volume.

The springback ($\varepsilon_{sb}$%) is defined:

(5) $$\varepsilon_{sb}\left(\%\right)=\frac{h_{ac1}-h_{ac0}}{h_{bc}}\times 100$$

where $h_{bc}$ is the initial height/thickness of the sandwich structure before compression; $h_{ac0}$ denotes the height of the sandwich under compression loading, which was obtained from the force-displacement data of the compression test; and $h_{ac1}$ denotes the specimen’s height after compression loading was removed. Springback ($\varepsilon_{sb}$) also means the temporary deformation ratio (%) according to the initial thickness/height of the sample. The application of these characteristic compression properties is discussed in Section 3.2.

Thermal analysis was performed with Setaram Labsys Evo (Setaram, Adress: 28 Av. Barthélémy Thimonnier, 69300 Caluire-et-Cuire, France) simultaneous differential scanning calorimetry and thermogravimetry under an air atmosphere with 20 mL/min gas flow and 10 °C/min for DCP, EVA, and dissolved EVA in toluene (TEVA), and 1 °C/min heating rate for AZD and its mixture with ZnO in order to optimize the in situ foaming process. The analysis of EVA copolymer chemical structure after and before foaming was performed using a Thermo Scientific Nicolet iS20 FT-IR Fourier-transform infrared spectrometer (FT-IR).(Thermo Fisher Scientific Inc., Waltham, MA, USA).

Investigation of the samples’ morphology and failure mechanism was carried out using a HITACHI SU 1510 scanning electron microscope (Tokyo, Japan) with a tungsten filament to samples obtained both after and before the compression test. Samples were cut by an abrasive cutting machine and coated with gold–palladium (for EVA-filled HC) via sputter before imaging.

## 3. Results and Discussion

AHC structures are generally manufactured with the adhesive bonding method [33]. These structures are generally manufactured from Al 3000 and 5000 alloys and have a service temperature of 177 °C [33]. Therefore, the foaming temperature of the polymer should not exceed the service temperature of the AHC to prevent the debonding of nodes. Accordingly, the in situ foaming temperature was optimized via thermal analysis. The production of polymer foam with desired parameters depended on the concentration of additives and their activation temperature. To understand the foaming process mechanism of the AZD/ZnO mixture, the thermal behavior of EVA dissolved in toluene (TEVA), DCP, AZD, and AZD/ZnO mixtures were investigated under air between RT and 250 °C. Compression tests were performed for three EVA-filled AHC sandwich samples with different cell sizes. Their average values were used to evaluate their performance.

### 3.1. Production Optimization of Polymer Foam

**Figure 1:**
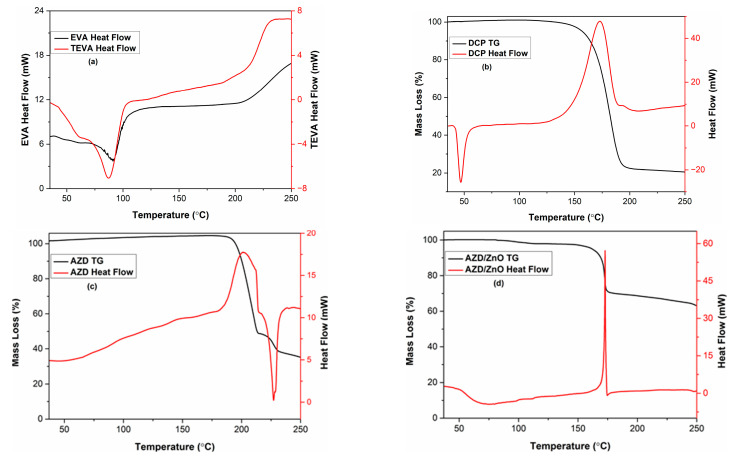
(**a**) DSC of EVA and TEVA. (**b**) DSC and mass loss curves of DCP (**c**) of AZD and (**d**) of AZD:ZnO (1:1).

Figure 1a presents DSC curves of EVA and TEVA. The two endothermic peaks at 60 °C and 87 °C indicate the existence of two crystalline forms in EVA [38]. The lowest one indicates the crystal imperfection in the polymer matrix, while the second is related to the melting of the thicker crystalline form [38,39]. Figure 1a (EVA-TEVA) also shows that the molecular structure of EVA was not changed when dissolved in toluene. Thus, the solvent casting method is suitable to introduce chemical agents within the EVA mixture without any thermal alteration.

Figure 1b depicts the heat flow and TG curves of DCP used for thermochemical crosslinking agent. The endothermic peak indicates that the melting of DCP occurs at 47 °C. Then, the exothermic reaction suddenly starts at 154 °C and lasts up to 182 °C. During this thermal decomposition stage, DCP generates free radicals that abstract hydrogen atoms to produce alkyl radicals [40].

Figure 1c,d show the thermogram and DSC curves of AZD and AZD:ZnO (1:1) mixture, respectively. The AZD without ZnO started to decompose via an exothermic reaction at nearly 175 °C and accelerated at 205 °C, as seen in Figure 1c. During the decomposition process, solid and gaseous products occurred from the AZD [41]. ZnO triggered an abrupt degradation of AZD at 175 °C, inducing a high heat flow and a high gas volume release (Figure 1d). In other words, ZnO decreases the activation energy for AZD decomposition [42]. The baseline slope of the DSC curve at temperatures above 150 °C could be related to the change in the AZD heat transfer due to the liberation of gaseous and degradation of the solid products [43]. The AZD mass loss after the exothermic peak was about 54%, followed by an endothermic peak indicating possible structural recombination above 210 °C involving an additional 14% mass loss. With the AZD:ZnO mixture, as expected, the mass loss was half-reduced at 175 °C but not followed with an important endothermic reaction. An additional 7% mass loss is detected at the end of the thermogravimetric analysis indicating that the possible structural recombination is triggered but slowed down after AZD activation at the lowest temperature. Finally, since the total AZD loss is comparable for both samples, it can be considered that ZnO is not chemically present in the product of activated AZD. Accordingly, the temperature of the in situ foaming of EVA was selected as 160 °C for 1 h.

**Figure 2:**
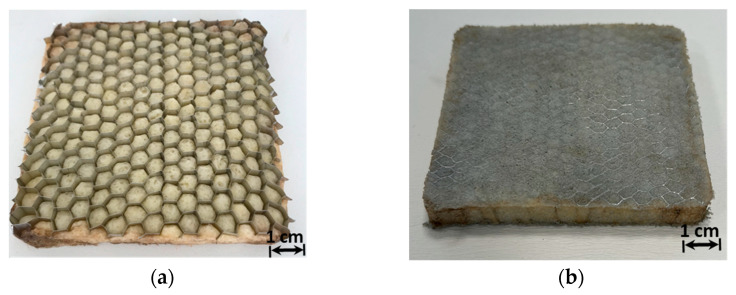
(**a**) Hybrid sample before the foaming process (**b**) the in situ EVA-foamed AHC core.

The foaming process consists successively of mechanical reinforcement of the melted EVA via DCP decomposition, followed by the decomposition of AZD, which involves the spreading of gas bubbles within the strengthened mixture. Each of the two steps strongly depends on the other to prevent cell coalescence and provide a sufficient expansion. In addition, the process time and proportion of the additives played a significant role in the foaming process. In this study, 1% DCP, 3% AZD, and 3% ZnO mixtures prepared with EVA (Figure 2a) were in situ foamed at 160 °C for 1 h in the AHC structure (Figure 2b). The weight and volume values of the AHC and the polymer mixture were measured during the production of hybrid materials. The 0.941 g/cm^3^ density EVA as raw material decreased to about 0.38 g/cm^3^ for the mixture after the in situ foaming process according to these calculations.

**Figure 3:**
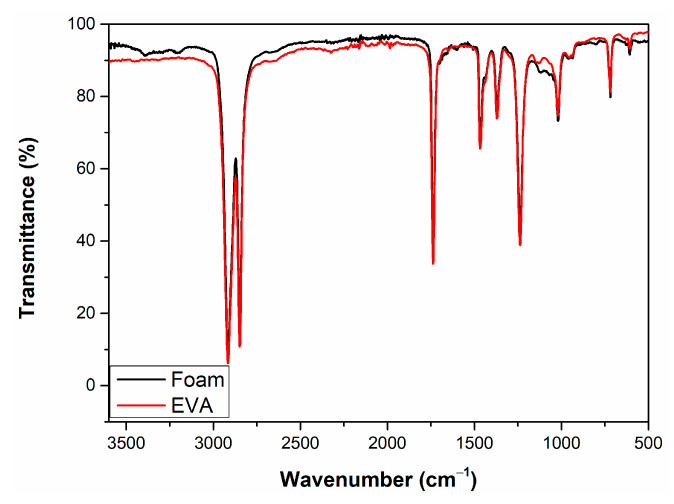
FT-IR spectra of raw EVA granules and EVA foam.

In order to control the evolution of EVA copolymer chemical structure after foaming, the FT-IR spectrum of raw EVA was compared with the foam (Figure 3). Both spectra are almost similar, except for differences in the two regions. Two weak peaks at about 3300 and 3200 cm^−1^ were not observed on the raw EVA granules that appeared after foaming. These peaks are attributed to the hydroxyl and peroxide functional groups possibly resulting from the crosslinking and foaming reaction [44]. The second region is at about 1150 cm^−1^ ascribed to the stretching of ester (-C-O-C-) bonding, where the EVA granule weak peak disappears after the EVA foam preparation [45,46]. These results demonstrate that the foaming is not modifying the fundamental structure of EVA copolymer; it only involves the formation of new bonding around the oxygen functional groups.

**Figure 4:**
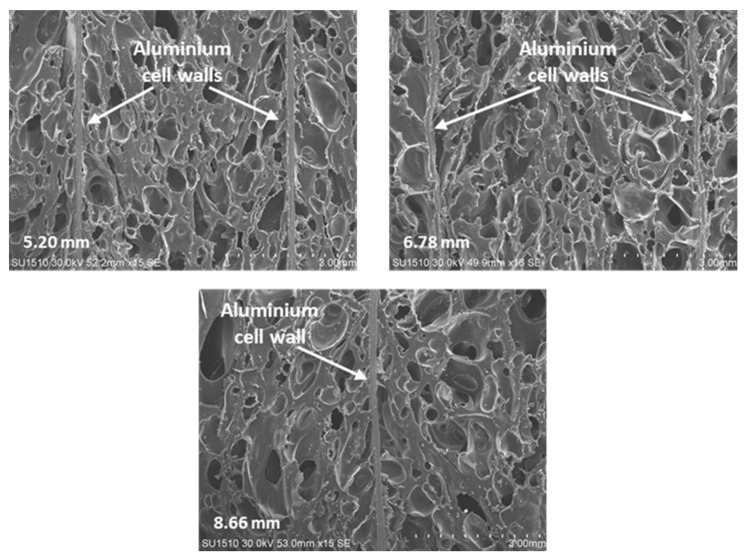
SEM images of the cross-sectional areas of the EVA-filled AHC core with 5.20/6.78/8.66 mm cell sizes.

Figure 4 shows the cross-section area of the microstructure of the in situ EVA-foam-filled AHC structure with different cell sizes of 5.20, 6.78, and 8.66 mm, respectively. EVA expanded between the aluminum cell walls without any significant shrinkage gap via in situ foaming method. Additionally, the EVA foam mainly comprised a closed cell structure, which affected the compression response of the hybrid sandwich panel as described in Section 3.2.

### 3.2. Compression Behavior of Sandwich Panels

The stress–strain curve obtained from the compression test of a typical cellular material consists of three distinct regions. The first one is the linear elastic region. The nominal stress increases almost linearly with increasing strain up to the first maximum stress and ends with the buckling of the cell walls [8]. The collapse initiates at the first maximum stress, which also indicates the yield strength (plastic deformation) of cellular materials. The strain value corresponding to the first peak stress is called the collapse initiation strain. It describes the strain value at which the plateau region begins, and it is the characteristic of cellular materials. The second stage is the plateau region where the stress is nearly constant with increasing strain. Large plastic deformations occur in this region, and the material absorbs most of the energy [47]. Therefore, the plateau stress determines the energy absorption capacity of the material [37]. The third characteristic region is the densification stage, where the cell walls contact each other. At this stage, the nominal stress increased sharply with strain. Cells come too close to each other, causing the structure to densify and increase the stiffness of the material [8,13]. The strain value, which represents the end of the plateau region and the transition to the densification region, is called the densification initiation strain (onset densification strain) [36]. This strain value can be determined by the energy efficiency method, as previously mentioned in the literature [34,35,36].

**Figure 5:**
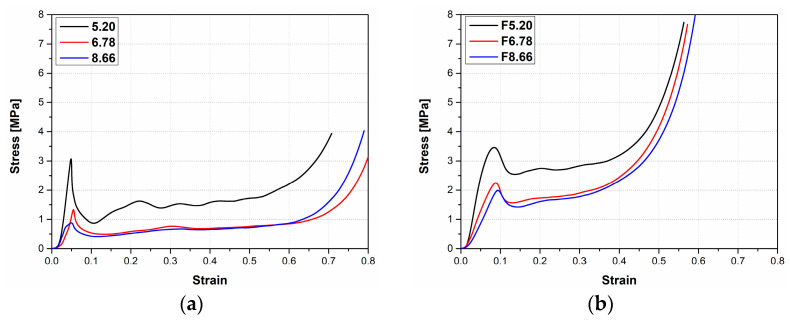
Stress–strain curves of sandwich structures with different cell sizes of aluminum HC: (**a**) unfilled aluminum HC; (**b**) EVA-filled HC.

Figure 5a,b gives our study’s stress–strain curves for sandwich panels of unfilled and EVA-foam-filled AHCs with various cell sizes. As can be seen in these figures, the stress–strain curves from the panels’ compression tests panels consist of three regions: (i) linear elastic region; (ii) plateau region; and (iii) densification region, similar to the compression behavior of typical cellular materials. In the elastic region, compressed aluminum cell walls undergo local deformation as buckling at a certain stress value (peak stress) [8,13,48,49]. Afterward, stress showed a decreasing trend called post-yield softening. In Figure 5a, the peak stress of the sandwich panels increased as the cell size of AHC decreased from 8.66 mm to 5.20 mm. The same trend was also seen in the stress–strain curves of EVA-foam-filled HC sandwich panels (Figure 5b). In both unfilled and EVA-filled samples, the highest mechanical properties are observed with the lowest cell-sized AHC (5.20 mm). However, the plateau region is shorter for EVA-filled specimens, indicating that densification starts at low strain values.

**Figure 6:**
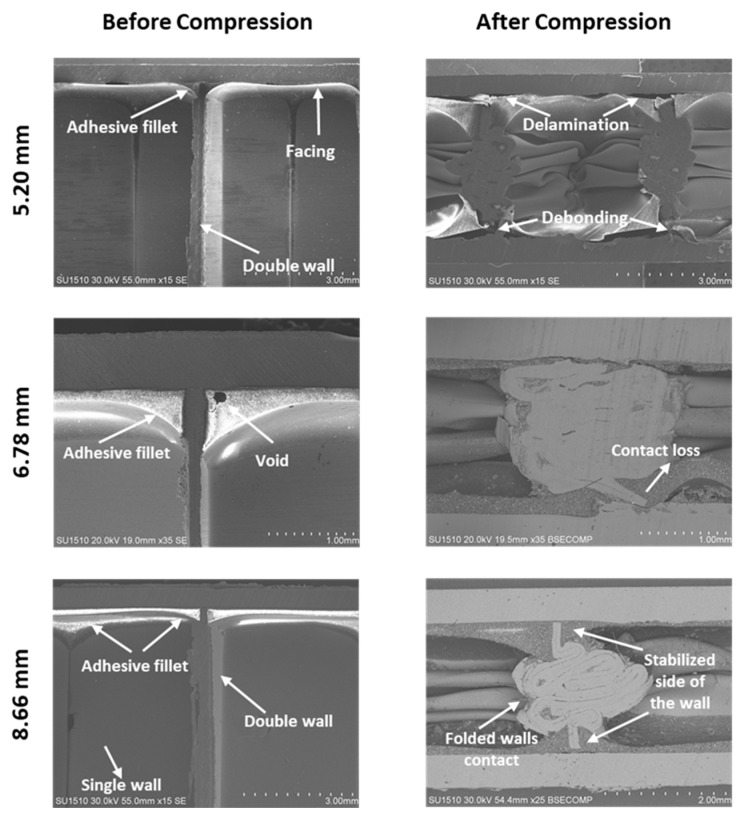
Cross-sectional electron images of the empty aluminum honeycomb with 5.20 mm, 6.78 mm, and 8.66 mm cell sizes (top the bottom) before compression and after compression (left to right).

Microstructural observations of the unfilled honeycomb sandwich panels before and after compression test were investigated by SEM with both secondary and backscattered electrons. The cross-sections of the unfilled AHC sandwich panels with 5.20 mm, 6.78, and 8.66 core cell sizes before compression and after compression are presented in Figure 6. Some critical and characteristic failure points of the sandwich panel were marked on the images. The adhesive applied for bonding between the facing sheet and the AHC core flowed throughout the facing plates with the effect of the curing temperature. During this process, the adhesive accumulated at the junction of the aluminum core and face sheets creates menisci-shaped fillets [50]. The gasses released during the adhesive polycondensation are trapped within the AHC cells increasing the inside pressure and contributing to the formation of these menisci. The effect of the fillet characteristics, such as size, height, and depth, on the panel mechanical properties was complex [51]; however, their existence provided strength enhancement of the sandwich panels, as previously described by Bitzer [33] and Paik et al. [52]. Therefore, the collapse strength of AHC with smaller cell sizes is enhanced due to the increase in adhesive bonding points with the facing sheet (Figure 5a).

Nevertheless, the interfacial bonding failures in the forms of debonding or delamination between the aluminum core and the facing sheet occur after a sufficiently high out-of-plane compression loading (Figure 6). However, the structure still has the capability of transferring the stress via fillet. This phenomenon could also be seen in the cross-section image of sandwich panel with 8.66 mm cell-sized AHC core. The cell walls folded progressively during the plateau stage and ended as completely folded with the contact of walls at the end of the densification stage. However, the double-wall side of the aluminum core, which was adjacent to the facing sheet, was prevented from collapsing. This phenomenon was considered during the investigation of the failure mechanism of the foam-filled and the unfilled AHC sandwich structures [53,54].

**Figure 7:**
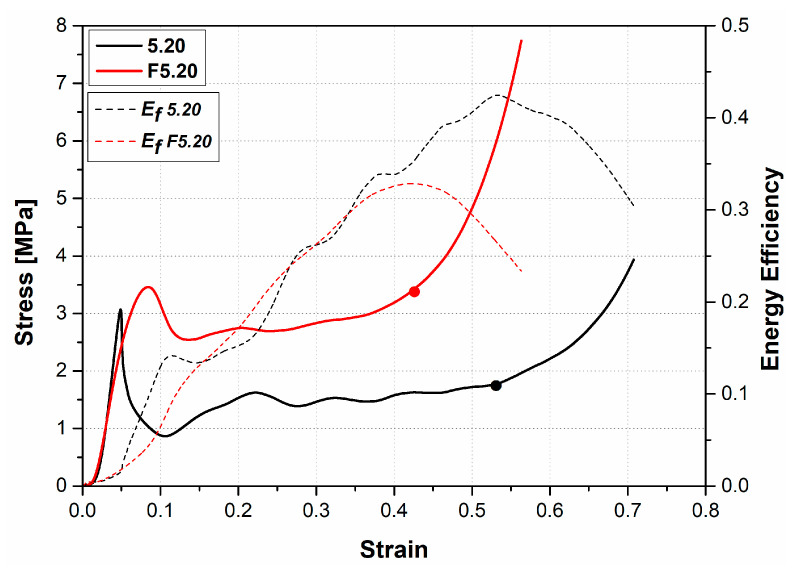
Stress–strain and energy efficiency curves of the unfilled and EVA-foam-filled aluminum honeycomb panel with 5.20 mm core cell size.

The stress–strain and energy efficiency curves of the EVA-filled and the unfilled AHC panels with 5.20 mm cell size are presented in Figure 7. The maximum point of the energy efficiency curve indicates the densification strain. The left vertical axis shows the stress values. At first, the stress increased almost linearly with increasing strain for both specimens associated with axial waves occurring on the cell walls due to the elastic response. When the stress reached a certain value, the materials had lost their stiffness, and right after, this decreased with increasing strain. Ashby defines this decrement as post-yield softening [55]. An abrupt decrease in the stress is observed with the unfilled AHC sandwich panel, while it is delayed with the EVA-foam-filled structure showing the beneficial contribution of the polymer on the compression strength performance (Figure 7). The cell walls of the aluminum were bent and buckled at the local maximum point and started a creating fold, which is the main deformation in thin-wall-type structures [48,55,56]. Following buckling, the structure started to carry a low load due to stress relaxation and this led to a minimum local point formation.

The stress decreased nearly from 3 MPa to 0.8 MPa for the unfilled honeycomb and from 3.4 MPa to 2.51 MPa for the EVA-foam-filled honeycomb sandwich panel. The fold walls within the unfilled AHC contribute independently toward the out-of-plane compression. The presence of EVA involves two beneficial effects: (i) physical contact between them allowing load transfer; and (ii) an additional strength tightly related to the foam density. Furthermore, in the plateau stage of the curve, further plastic hinges and folding formation cause stress oscillations. The latter decrease significantly for the EVA-foam-filled AHC compared with the unfilled samples. Consequently, foam-filled panels are more rigid and stable under compression loads.

**Figure 8:**
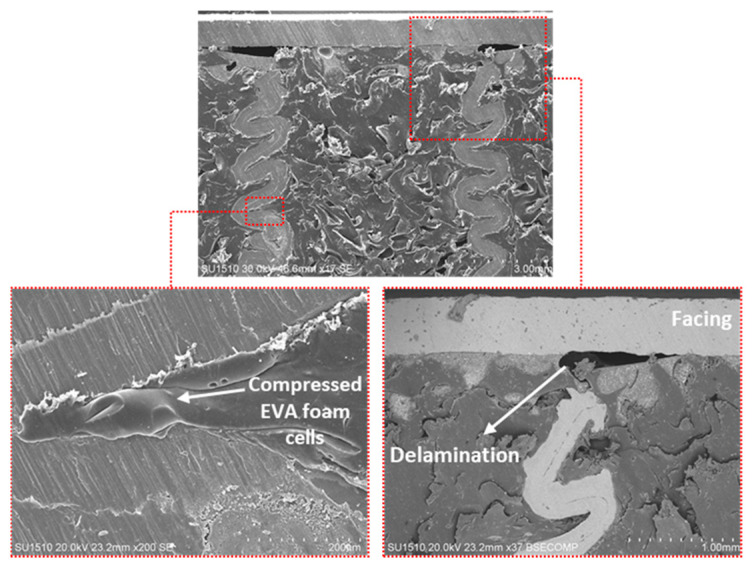
Electron images of the cross-section area of the EVA-foam-filled HC with 5.20 mm cell size after the out-of-plane compression test.

Figure 8 shows electron images of the EVA-foam-filled aluminum HC with 5.20 mm cell size after the out-of-plane compression test. Folded aluminum cell walls are not in contact with each other due to the existence of EVA foam between the cell walls. Although some EVA foam cells were compressed by the peak sides of the folded Al walls, some remained with gas inside and preserved their shape. Gas inside the EVA foam cells was first squeezed out in closed cells or diffused from open cells under loading [57]. After that, some air was withdrawn back to the cells again and the EVA foam between the aluminum cell walls was recovered up to somewhat-strain level when the load was removed due to the viscoelastic nature of the foam. The EVA-foam-filled AHC sandwich structure still had energy-absorbing capability with the foam cells with gas and recovered strain even after compression loads at high levels. However, delamination failure also occurred at the junction of the rigid aluminum double walls and facing sheet because of the strain recovery behavior (Figure 8).

During the out-of-plane compression test, delamination was observed between the AHC wall extremity and the facing sheet (Figure 8). However, the adhesion between the EVA-foam and the aluminum surfaces (AHC wall and facing sheet) was maintained and contact between the folded aluminum cell walls was prevented. Thus, there is a synergetic effect between the EVA foam and the AHC toward compression forces.

**Figure 9:**
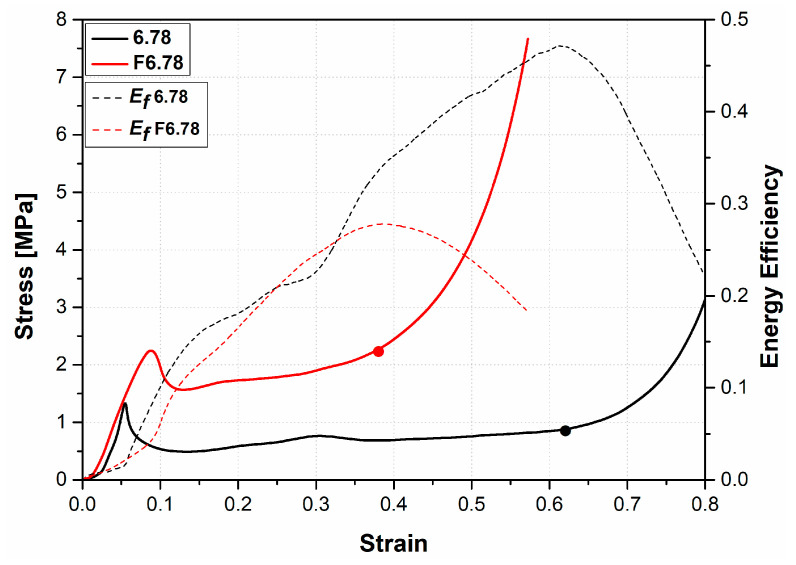
Stress–strain and energy efficiency curves of empty and EVA-foam-filled aluminum honeycomb panel with 6.78 mm core cell size.

**Figure 10:**
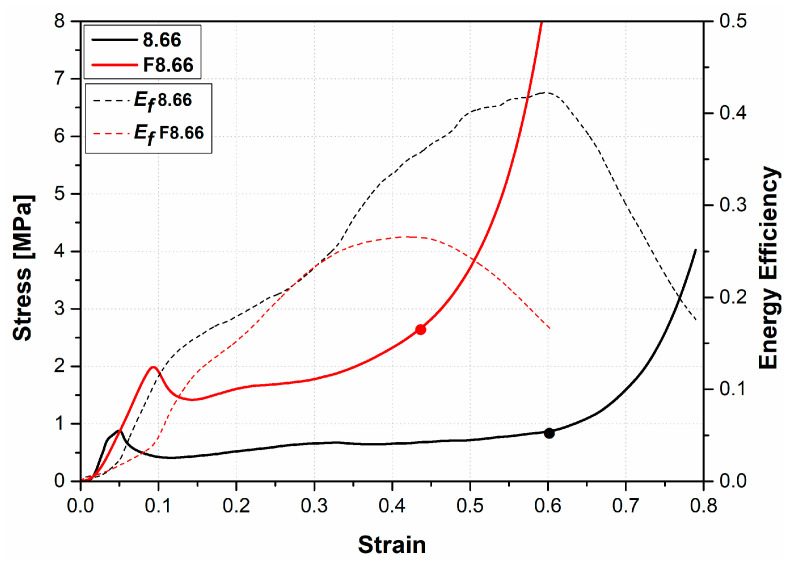
Stress–strain and energy efficiency curves of empty and EVA-foam-filled aluminum honeycomb panel with 8.66 mm core cell size.

The highest compression strength of the EVA-foam-filled sandwich panels was also observed when increasing the AHC cell size (Figure 9 and Figure 10). Mahmoudabadi et al. [21] obtained similar results. However, three main differences appear: (i) the energy absorbed is lowest; (ii) the difference between the unfilled and filled specimen is increasing; and (iii) the stress oscillations are reduced. Therefore, the damage mechanism in the stretch-dominated aluminum cell walls was primary in the general deformation mode for foam-filled samples. Furthermore, the contribution of EVA foam filler to the stiffness and energy-absorbing capacity became more evident as the cell size of the AHC increased.

Figure 9 and Figure 10 also show the energy efficiency curves. The maximum values of these curves indicate the densification strain. It was also deduced that the densification strain of the structure decreased when the aluminum honeycomb was filled with EVA foam.

**Figure 11:**
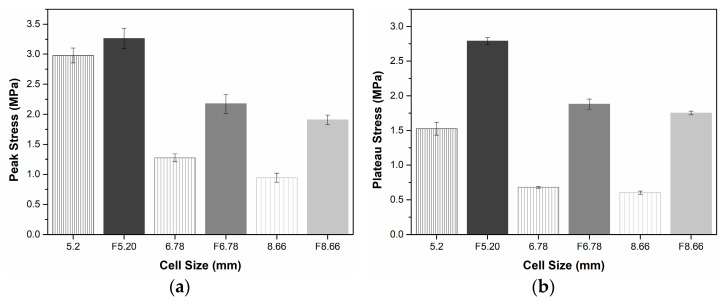

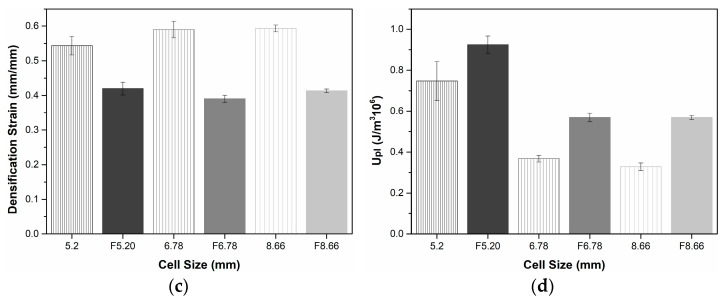
Compression characteristic criteria of empty and foam-filled aluminum HC with cell sizes 5.20 mm, 6.78 mm, and 8.66 mm: (**a**) peak stress; (**b**) plateau stress; (**c**) densification strain; (**d**) absorbed energy per unit volume.

Compression characteristic criteria of empty and foam-filled aluminum honeycomb with cell sizes 5.20 mm, 6.78 mm, and 8.66 mm are given in Figure 11 to compare both the cell size and filling effect. As can be seen from Figure 11a, the peak stress of empty HC with cell size of 5.20mm increased from 2.98 MPa to 3.26 MPa when filled with EVA foam, increasing by 9.56%. In contrast to the smaller cell size (5.20 mm), the enhancement of the peak stress by EVA-filling was significantly evident as the cell size increased. While the peak stress of the aluminum HC panel with 6.78 mm cell size was increased by 70.78%, the peak stress of the 8.66 mm cell size honeycomb was doubled and increased by 102% when filled with EVA. It was also determined that the peak stress increased as the cell size decreased from an examination of the peak stress trend of both filled and empty aluminum honeycomb panels. Thus, the observation of a similar trend in both the empty and filled samples indicated that the first local deformation mechanism at the end of the elastic region was dominated first by the buckling of AHC.

Plateau stresses of the empty and EVA-filled HC structures obtained from the stress–strain curves between the collapse initiation and onset densification strain are given in Figure 11b. In contrast to the low increase in the peak stress of HC with 5.20 mm cell size (see Figure 11a), the plateau stress increased by 83% when filled with EVA foam. The partial restriction of yield softening after critical strain through the filling EVA foam provided the occurrence of this difference between the two stresses. Additionally, progressively plastic buckling of the cell walls required more stress because the cells of aluminum were full of EVA foam. From Figure 11b, this relation was also observed for honeycombs with 6.78 mm and 8.66 mm cell sizes. While the plateau stress increased by 176% when EVA was filled for a honeycomb with a 6.78 mm cell size, the stress of the honeycomb with a larger cell size (8.66 mm) increased by 190%. The significant enhancement of plateau stresses of EVA-filled sandwich structures indicated that the aluminum cell walls and EVA foam have great compatibility and provide a strong interaction.

On the other hand, as seen in Figure 11b, the plateau stress decreased as the cell size increased for both empty and EVA-filled honeycombs considering the geometrical design parameter. Several authors have described before the relationship between the enhancement of the plateau stress and increasing the ratio of the wall thickness and edge length (t/l) [47,58,59]. In this study, the cell size was represented as inversely proportional to the t/l ratio due to the use of the same aluminum foil thickness (t) for all sandwich structures. Decreasing the cell size of aluminum honeycombs increased the volume of the aluminum material for the same specimen size. Therefore, the sandwich structure behaved more rigidly during the folding mechanism due to the high volume of aluminum and provided enhancement of the plateau stress [60]. The same trend was also observed for EVA-filled honeycombs despite their higher strength.

Figure 11c shows the onset densification strain of the sandwich structures for empty and EVA-filled HC. The densification initiation strain of aluminum honeycombs with cell sizes of 5.20 mm decreased by 22%, and decreased by 33% and 30% for 6.78 mm and 8.66 mm, respectively, when filled with EVA foam. The drop in the densification strain indicated that the plateau stage ended earlier in comparison to the HC without EVA foam. Similar results were also obtained from PU or EPP foam-filled honeycombs according to Nia et al. [14] and Liu et al. [23], respectively. However, the total absorbed energy (per unit volume) given in Figure 11d of the structures is enhanced due to the correlation between the increase in plateau stress and the decrease in onset densification strain. In Figure 11d, the absorbed energy per unit volume by the sandwich structure increased by 23, 54, and 73% for EVA-filled honeycombs with 5.20 mm, 6.78 mm, and 8.66 mm cell size, respectively.

### 3.3. Post-Compression Behavior of the EVA-Filled Panels

**Figure 12:**
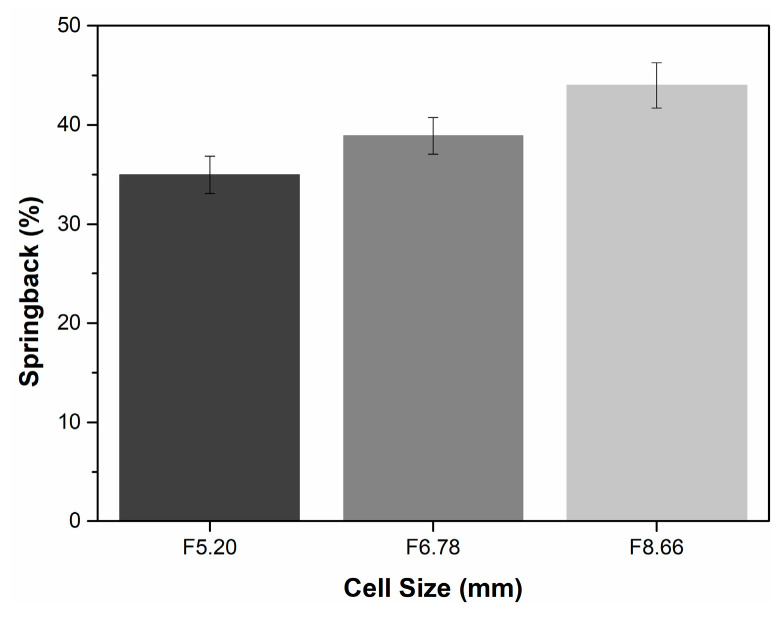
Springback (%) of EVA-foam-filled aluminum honeycomb sandwich panel with different honeycomb cell sizes after compression loading removed.

Finally, the EVA-foam-filled aluminum HC sandwich panel thickness recovered at a particular strain level when the compression loading was removed. This phenomenon clearly indicated that the use of a thermoplastic elastomer filler for a relatively rigid aluminum material ensured characteristic springback behavior in the structure. Figure 12 represents the springback (%) of the EVA-foam-filled structures of Al cores with different HC cell sizes. As can be seen in Figure 12, the springback percentage increased from 35% to 44% as the HC cell size increased from 5.20 mm to 8.66 mm. These recovery rates were related to relatively dependent parameters. One of these was that increasing the density of aluminum rigid ribbons as the aluminum HC cell size decreased partially prevented the springback of compressed EVA foam. The other was that the increased aluminum honeycomb cell size allowed more continuous EVA-foam cells and provided more gas drawn back per unit area.

**Figure 13:**
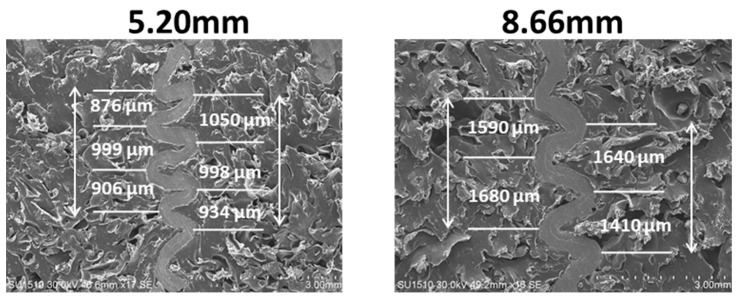
Distances between the peaks of the folded double walls of EVA-foam-filled aluminum HC structure with 5.20mm and 8.66mm cell sizes.

Springback phenomenon was also observed in microscopic investigations of EVA-foam-filled honeycomb structures after the compression test. Cross-sectional areas of the folded double wall of foam-filled HC with 5.20 mm and 8.66 mm cell sizes are given in Figure 13. According to Figure 13, a measurement of each distance between peaks of double wall folds toward the axial direction indicated that the distance increased due to increasing springback (%). The EVA foam triggered more recovery of the compressed strain as the cell size of aluminum HC increased due to the relatively easier movement of the squeezed-out gas inside the EVA cells.

**Figure 14:**
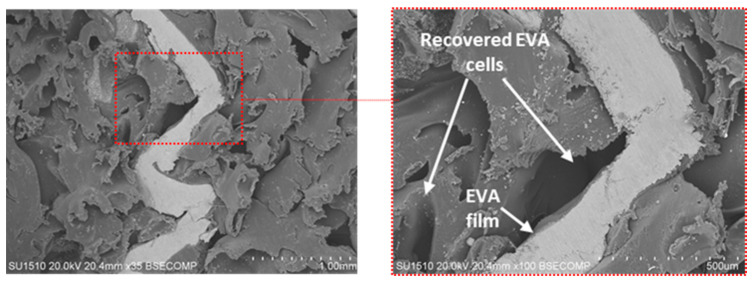
Recovered EVA cells after compression and interaction between EVA cells and aluminum cell walls.

Ultimately, given the entire compression properties as well as the failure mechanism of the panel, the compressive response of the structure was strongly controlled by the interaction between the aluminum cell walls and the EVA foam. If the polymer foam were not sufficiently interacting with aluminum cell walls, it would have resulted in more permanent and sudden failure due to rupture after compression. At this point, the in situ foaming process for filling the HC structure provided a strong interaction between the aluminum cell walls and EVA. Figure 14 shows the recovered EVA cells after compression and interaction between the EVA cell and aluminum cell wall. As seen in Figure 14, which provides a compositional contrast between the polymer and metal (BSE image), melted EVA was stretched to an aluminum cell wall as a film by the formation of bubbles during the foaming process. Moreover, the recovered EVA foam cells marked in Figure 14 indicated that the structure still has the energy-absorbing capability through the viscoelastic nature of the foam even after high compression loading conditions.

## 4. Conclusions

This study determined the compression behavior of novel in situ EVA-foam-filled and empty aluminum hexagonal honeycomb sandwiches under quasistatic out-of-plane loading conditions. Scanning electron microscopy was used to investigate the failure mechanisms in detail. According to the optimization of the foaming process, microstructural observations, and compression test data, the following results were obtained.

The EVA foaming process is suitable for in situ foam-filling into honeycomb without damaging aluminum cell walls. In situ filling of foam provides a strong interaction between EVA and aluminum cell walls due to stretched EVA during the foaming process.

The general stress–strain pattern was dominated by the buckling and folding of the aluminum walls in both empty and EVA-foam-filled HC. Buckling at peak stress comprised several strain values, and the difference between local maximum and minimum stress points decreased in EVA-foam-filled honeycombs.

Plateau stress increased by 83%, 176%, and 190% in the honeycomb with 5.20, 6.78, and 8.66 mm cell sizes, respectively. Absorbed energy per unit volume of sandwiches was also significantly enhanced with EVA foam filling for out-of-plane loading.

The filling of EVA foam led to a decrease in the densification strain of 22%, 33%, and 30% for 5.20, 6.78, and 8.66 mm cell size HC, respectively. An EVA-foamed sandwich with an 8.66mm aluminum cell recovered 44% of the initial height after the compression load was removed.

Since the sandwich panels maintained their structural integrity largely after compression loadings, the structure still had an energy-absorbing capacity due to the springback.

## Figures and Tables

**Table 1:** Properties of the aluminum core material.

Specimen Code *	Cell Size (mm)	Foil Thickness (mm)	Core Thickness (mm)	Density (kg/m^3^)
5.20	>5.20	0.05	10	69.76
6.78	>6.78	0.05	10	53.50
8.66	>8.66	0.05	10	41.89

* F5.20, F6.78, and F8.66 were used as the nomenclature of filled specimens.

## Data Availability

Data are unavailable due to privacy or ethical restrictions.
